# Ionization in the Treatment of Cicatrices of the Face

**Published:** 1918-10

**Authors:** 


### IONIZATION IN THE TREATMENT OF CICATRICES OF THE FACE

(Translations specially made and condensed for the *Dental Record.*)

*Technique.*—A continuous current of 220 volts is used for this work; the current should be a weak one (ten milliampères) as the passage of potassium iodide produces a much more rapid effect on cicatrical tissue in these circumstances. The solution should be one per cent. in distilled water.

The positive electrode is generally five by four centimeters square, but its size varies with the extent of the cicatrix. For the face the electrodes are very close together. The negative pole is placed on the cicatrix to be treated, the positive pole is generally placed under the chin. The positive pole is moistened with the KI solution. Treatment should be given every day for twenty to twenty-five minutes for three or four weeks. It may be necessary to prolong the treatment for several months in obstinate cases.

*Clinical Phenomena.*—(1) Smooth cicatrices with disappearance of the dermis.

(2) Fibrous cicatrices with thickening of the cicatrical tissues.

(3) Cicatrices adherent to the bone.

(4) Parchment-like cicatrices resulting from extensive burns.

1. Smooth cicatrices are smooth rose colour, supple, and have a certain sensibility when touched, and to thermal changes.

2. Fibrous cicatrices appear like a stretched cord on the surface of the skin, but raised above it; generally mammilated, white in colour, and may be several millimetres thick. They impede the movements of the facial muscles. Circulation of the blood does not seem to exist.

3. Cicatrices adherent to the bone are almost always in the form of a cavity with a smooth base. The tissue is joined to the bone and can not be displaced with the fingers.

4. In this class the dermis has entirely disappeared; the colour is copper red. Movement of the muscles beneath is very limited. The face appears drawn.

*Treatment.*—The same results of cure have not been obtained in all cases. In smooth cicatrices one first notices a change of colour. From a violet red the skin becomes pink, and then white, to attain in a few days the normal colour of the skin. This result is obtained in from two to three weeks.

Fibrous cicatrices require much longer treatment. At the beginning induration disappears and the cicatrix becomes soft; the bands disappear and the softer condition is apparent at the end of a month's treatment. Throughout its length it becomes detached from the deeper layer and takes on the appearance of the ordinary skin. The circulation of the blood is resumed and the tissue appears to be vascularized.

In the case of adherent cicatrices the results are almost always obtained in a month; adhesion to the bone entirely disappears and the superficial layers can easily be moved over it.

A unique case of a cicatrix due to a generalized burn required much longer treatment, but at the end of six weeks the parchment-like tissue could be pressed in; trophic and vascular trouble had disappeared in part.

The patient could open his mouth without fear of tearing the commissures of the lips; he could also laugh; at the end of ten weeks small pigmented areas made their appearance; these gradually enlarged and united with one another, presenting the aspect of ordinary skin.

Of sixty cases during a year, forty-three were fibrous cicatrices and thirty-seven of these were completely successful. Failure was due to the large amount of cicatrical tissue, the bands were fifteen to twenty cm. long and two to three cm. wide. They involved the cheek and a part of the neck. Treatment was of value though, because the cicatrix was detached from the deep layer, and this enabled Dr. Pont to easily remove them. Massage and ionization were resumed after the wound had healed and the patients presented a supple cicatrix of good appearance.

Case 1. Patient aged forty-two, wounded 15th March, 1915.

Received for treatment 31st March, 1917, after he had undergone plastic operations in other hospitals. Presented a fibrous cicatrix stretching from the right horizontal ramus to the symphysis. Another cicatrix stretched from the middle of the lower lip to the left cheek and was eight cm. long. The opening of the mouth was four cm. long. The fleshy mass of the chin was adherent to the deep layer. The teeth could receive no attention whatsoever during the last four months, because the patient could not open his mouth sufficiently. After four months' treatment the adhesion to the bone had disappeared; the commissures had become supple; the patient could whistle and the fleshy portion of the chin was easily displaced by pressure. Dr. Pont enlarged the labial commissures, the one on the right by ten mm. and that on the left by fifteen mm. without difficulty. The complete softening was obtained a month later by means of the lip dilator.

Case 2. Age thirty-three. Wounded 9th November, 1917, received for treatment 16th November, 1917. Injury, fracture of the right mandible with loss of bone from the angle.

On the 29th November there was no longer any discharge of pus (curetting had been performed previously). A cicatrix adherent to the bone of the horizontal ramus formed. After a fortnight's treatment with massage and ionization the adhesions disappeared; the skin could be displaced under the finger and it had no contact with the bone.

Case 3. Age twenty-three. Burnt in a fall from a burning aeroplane, 25th February, 1917. Entered the hospital 26th May, 1917, having previously been treated with ambrine. There was refraction of all the integuments of the face; deformity from the aesthetic point of view, exopthalmia, deformity of the orbital arches and of the nose; loss of parts of the eyelids. Atresia of the lips; occlusion is impossible. In October, 1917, the following results had been obtained by ionization, which treatment was given from the time he was taken in. The mouth could be closed; the lower eyelids are softened; the skin has become smooth and soft; pigmentation has reappeared in places and the skin is growing again. The treatment has already lasted several months and is being continued.—*La Restauration Maxillo-Faciale,* February, 1918.

